# Vitamin B12 Deficiency in Patients With Diabetic Peripheral Neuropathy: A Hospital-Based Cross-Sectional Study

**DOI:** 10.7759/cureus.87490

**Published:** 2025-07-07

**Authors:** Tariq Hayat, Ramshaw Waqar, Salman Farsi, Sakhawat Ali, Muhammad Asim, Umar Farooq

**Affiliations:** 1 Acute Internal Medicine, Lady Reading Hospital Peshawar, Peshawar, PAK; 2 Acute Internal Medicine, Russells Hall Hospital, Dudley, GBR; 3 General Medicine, Ayub Teaching Hospital, Abbottabad, PAK; 4 Medicine, Ayub Medical College, Abbottabad, PAK; 5 Medicine, Lady Reading Hospital Peshawar, Peshawar, PAK

**Keywords:** cobalamin, diabetes complications, diabetes mellitus, diabetic neuropathy, nutritional deficiency

## Abstract

Background

Diabetic peripheral neuropathy (DPN) is a serious but potentially preventable complication of diabetes mellitus, particularly in individuals with vitamin B12 deficiency. Despite clinical guidelines, routine B12 screening remains inconsistent, and hospital-based data on deficiency patterns in DPN populations are scarce. This study evaluated the frequency and determinants of B12 deficiency among patients with DPN at a tertiary care center in Pakistan.

Materials and methods

We conducted a hospital-based cross-sectional study on 139 patients (ages 30-60 years old) with type 2 diabetes and confirmed DPN (Michigan Neuropathy Screening Instrument score ≥4) at Lady Reading Hospital, Peshawar (August 2022-March 2025). Patients taking prior B12 supplementation or with malabsorption disorders were excluded. We measured the serum B12 levels, defining the deficiency as less than 149 ng/L. We used a multivariable logistic regression model to identify the predictors of B12 deficiency, adjusting for age, gender, and diabetes duration.

Results

Vitamin B12 deficiency was prevalent in 48.2% (67/139) of participants. Moreover, it was significantly higher in older patients (54.7% in the 46-60 age group vs. 13.6% in the 30-45 age group, p < 0.001) and those with longer durations of diabetes (72.8% with >5 years vs. 13.8% with 1-5 years, p < 0.001). There was no statistically significant difference among genders (50.5% males vs. 42.9% females, p = 0.38). Analysis showed that being over 45 years old and having diabetes for more than 5 years are strong factors that predict vitamin B12 deficiency.

Conclusions

This study demonstrates a high burden of vitamin B12 deficiency among patients with DPN, which is strongly associated with age and duration of diabetes. Implementation of routine B12 screening protocols for patients with DPN, particularly those of old age or with prolonged duration of diabetes, may help prevent neurological deterioration in this vulnerable population.

## Introduction

Peripheral neuropathy (PN) comprises a diverse group of disorders characterized by dysfunction of the peripheral nervous system, often presenting with symptoms such as numbness, tingling, or burning sensations in the extremities [1]. While metabolic conditions remain the most common underlying cause of extremity neuropathic pain, PN can also result from a wide range of other clinical conditions [1].

Among its many etiologies, diabetes mellitus is associated with multiple potentially disabling or life-threatening complications [2]. One of these complications, diabetic PN (DPN), is the most prevalent and can lead to serious consequences [2]. Early recognition and assessment of peripheral polyneuropathy symptoms are essential to prevent the development of neuropathic foot ulcers, which are associated with increased morbidity and mortality [3]. Chronic hyperglycemia contributes to microvascular damage, impairing the delivery of oxygen and nutrients to nerves [4,5]. This process typically begins with damage to distal sensory and autonomic nerve fibers. It may progress proximally, resulting in a gradual loss of protective sensation in the feet and lower limbs. Asymmetric presentation is seen in approximately half of DPN cases. Without adequate preventive foot care, patients are at high risk of injury and subsequent complications due to insensate extremities [6].

Vitamin B12 (cobalamin) is a water-soluble vitamin primarily derived from animal-based dietary sources such as red meat, dairy products, and eggs [7]. Its absorption requires an intrinsic factor, a glycoprotein produced by gastric parietal cells, which facilitates B12 uptake in the terminal ileum. Once absorbed, B12 acts as a coenzyme in the synthesis of DNA, fatty acids, and myelin, making it essential for hematologic and neurologic function [8]. Despite substantial hepatic stores, deficiency may develop gradually in individuals with poor dietary intake, gastrointestinal malabsorption, or lack of intrinsic factors [9].

A study reported that 64% of patients with DPN were deficient in vitamin B12 [10]. This high prevalence raises concerns, especially in those receiving long-term metformin therapy, a first-line pharmacologic agent for type 2 diabetes mellitus. Emerging evidence has linked prolonged metformin use to reduced B12 levels, thereby contributing to or exacerbating neuropathic symptoms. In response to this, the American Diabetes Association and the American College of Endocrinology have recommended routine monitoring of vitamin B12 levels in patients with diabetes, particularly those on chronic metformin therapy [11].

Despite these recommendations, B12 screening practices remain inconsistent, especially in resource-limited settings. Therefore, this study was designed to assess the frequency of vitamin B12 deficiency among patients with DPN at a tertiary care hospital in Pakistan and to identify the associated clinical and demographic determinants.

## Materials and methods

Study design and setting

This was a hospital-based, cross-sectional analytical study conducted at the Department of General Medicine, Lady Reading Hospital, Peshawar, a major tertiary care teaching hospital in Khyber Pakhtunkhwa, Pakistan. The study was carried out from August 2022 to March 2025. This institution receives a high volume of patients from both urban and rural areas, providing a representative population of individuals with chronic diseases such as diabetes mellitus.

Sample size and sampling technique

A total of 139 patients were included in this study. The sample size was calculated using the World Health Organization sample size calculator. The parameters used for calculation included a 95% confidence interval (CI), an 8% absolute margin of error, and an anticipated prevalence of vitamin B12 deficiency of 64% among patients with DPN, based on findings from a previously published study [10]. Participants were recruited using a non-probability consecutive sampling technique, wherein all eligible patients presenting during the study period and meeting the inclusion criteria were invited to participate. Adult patients, both male and female, aged 30 to 60 years, with known type 2 diabetes mellitus for more than one year and diagnosed with PN based on the Michigan Neuropathy Screening Instrument (score ≥4) [12], were included. Patients with a history of gastrointestinal surgery, diagnosed with malabsorptive disorders, and current or prior use of vitamin B12 supplements were excluded.

Data collection procedure

All patients who met the eligibility criteria were enrolled after obtaining written informed consent. Ethical approval for the study was obtained from the Institutional Review Board of Lady Reading Hospital Peshawar. Confidentiality of participants was maintained throughout the study. Demographic and clinical data were recorded using a structured questionnaire. Neuropathy was confirmed using the Michigan Neuropathy Screening Instrument, which includes both a symptom score and a clinical examination component. For biochemical analysis, 5 mL of venous blood was drawn from each participant under aseptic conditions. The samples were immediately stored in sterile, vertically positioned collection tubes and frozen at −30°C. All samples were transported to the hospital laboratory and analyzed on the same day using an automated chemiluminescence immunoassay analyzer. Serum vitamin B12 levels were measured in ng/L. Vitamin B12 deficiency was defined as a serum level of ≤149 ng/L.

Statistical analysis

Data were entered and analyzed using SPSS Statistics version 25 (IBM Corp. Released 2017. IBM SPSS Statistics for Windows, Version 25.0. Armonk, NY: IBM Corp.). Continuous variables, such as age and duration of diabetes, were expressed as means and standard deviations (SD), while categorical variables, including gender, presence or absence of B12 deficiency, and duration categories, were summarized using frequencies and percentages. Vitamin B12 deficiency status was stratified by age group (30-45 years and 46-60 years), gender, and diabetes duration (1-5 years and >5 years). The chi-square test was applied to evaluate the statistical significance of associations between categorical variables. A p-value of ≤0.05 was considered statistically significant. To identify independent predictors of vitamin B12 deficiency, a multivariable logistic regression model was employed. Variables included in the model were age group, gender, and duration of diabetes. Adjusted odds ratios (AOR) with 95% CI were reported to estimate the strength of the association.

## Results

The study included 139 patients with DPN. The cohort had a mean age of 49.8 ± 5.1 years (range: 30-60 years) and a mean diabetes duration of 5.2 ± 1.9 years. Male participants predominated (69.8%, n = 97) compared to females (30.2%, n = 42), as shown in Table 1. Nearly half of the study population (48.2%, n = 67) exhibited vitamin B12 deficiency (≤149 ng/L), as shown in Table 1.

**Table 1:** Baseline characteristics (n = 139) SD: standard deviation

Variables	Variable categories	Mean ± SD or n (%)
Age (years)	-	49.8 ± 5.1
Gender	Male	97 (69.8%)
	Female	42 (30.2%)
Diabetes duration (years)	-	5.2 ± 1.9
Vitamin B12 deficiency	Yes	67 (48.2%)
	No	72 (51.8%)

Furthermore, upon stratification, the data demonstrate a strong age-dependent pattern, with patients aged 46-60 years showing significantly higher deficiency rates (54.7%) compared to younger patients (30-45 years: 13.6%; p < 0.001). Diabetes duration emerged as another critical factor, with patients having a duration of >5 years showing a 72.8% prevalence of deficiency, compared to 13.8% in those with a duration of one to five years (p < 0.001). Gender differences were not statistically significant (males: 50.5% vs. females: 42.9%, p = 0.407, as shown in Table 2).

**Table 2:** Data stratified by age group, gender, and duration of diabetes, with chi-square test analysis after stratification x^2^ = chi-square test * Chi-square test applied to assess association after data stratifying into age groups, gender, and diabetes duration

Variables	Variable’s strata	Categories	n (%)	X^2^	df	p-value
Age group	30-45 years	Deficient	3 (13.6%)	13.21	1	<0.001^*^
		Normal	19 (86.4%)			
	46-60 years	Deficient	64 (54.7%)			
		Normal	53 (45.3%)			
Gender stratification	Male	Deficient	49 (50.5%)	0.69	1	0.407^*^
		Normal	48 (49.5%)			
	Female	Deficient	18 (42.9%)			
		Normal	24 (57.1%)			
Diabetes duration stratification	1-5 years	Deficient	8 (13.8%)	37.82	1	<0.001^*^
		Normal	50 (86.2%)			
	>5 years	Deficient	59 (72.8%)			
		Normal	22 (27.2%)			

Moreover, on multivariable logistic regression analysis (Table 3), diabetes duration >5 years (aOR: 3.12, 95% CI: 1.89-5.15, p < 0.001) and age >45 years (aOR: 2.45, 95% CI: 1.52-3.94, p=0.002) are identified as independent predictors of B12 deficiency after adjustment for potential confounders.

**Table 3:** Multivariable logistic regression analysis of factors associated with vitamin B12 deficiency aOR: adjusted odds ratio, CI: confidence interval * Identified independent predictors of vitamin B12 deficiency using a multivariate logistic regression model

Predictors^*^	aOR	95% CI	Wald X^2^	p-value
Diabetes >5 years	3.12	1.89-5.15	18.53	<0.001
Age >45 years	2.45	1.52-3.94	12.21	0.002
Female gender	0.78	0.42-1.45	0.62	0.431

## Discussion

This study found vitamin B12 deficiency to be a common and clinically significant comorbidity in patients with DPN. The strong associations with disease duration (more than five years) and age (over 45) identify high-risk populations who would benefit most from targeted screening and intervention.

Our study reveals a clinically significant prevalence of vitamin B12 deficiency (48.2%) among patients with DPN, contributing to the growing body of evidence supporting this important metabolic association. While this is substantial, it is somewhat lower than the 64% reported by a previous study, although it falls within the wide range (5-40%) documented in the previous literature [13-16]. This variation likely reflects differences in study populations, diagnostic criteria, and sample size when interpreting these findings. The robust correlation we observed between vitamin B12 deficiency and longer diabetes duration (aOR 3.12, p < 0.001) strongly supports the existing pathophysiological understanding of metformin's interference with B12 absorption mechanisms in the terminal ileum [17-20]. This duration-dependent relationship suggests a cumulative effect, where each additional year of diabetes management may progressively increase the risk of deficiency. However, we had not investigated the use of metformin.

The absence of significant gender differences in B12 deficiency prevalence (males: 50.5% vs. females: 42.9%, p = 0.407) contrasts with some previous reports [21] and may reflect unique aspects of our study population, including dietary patterns, genetic factors, or healthcare utilization behaviors, as well as the small sample size. This finding suggests that screening protocols in similar populations should not be gender-targeted, though further research is needed to clarify the biological and social determinants of this observation. The relatively lower prevalence of DPN in our cohort compared to previous reports (39-50%) [22,23] may reflect differences in diagnostic methodology or population characteristics, highlighting the ongoing need for standardized diagnostic criteria in both clinical practice and research settings.

Several important clinical implications emerge from these findings. First, the high prevalence of B12 deficiency supports routine screening for all DPN patients, particularly those with longer diabetes duration or advanced age. Second, clinicians should maintain heightened vigilance for neurological symptoms in high-risk patients, recognizing that B12 deficiency may both exacerbate and mimic the manifestations of diabetic neuropathy. Third, consideration should be given to prophylactic B12 supplementation for patients initiating metformin therapy, especially those with additional risk factors. These recommendations are particularly relevant given the progressive nature of both B12 deficiency and DPN, where early intervention may prevent irreversible neurological damage.

The limitations of our study must be acknowledged, including its single-center design, which may limit generalizability, and its cross-sectional nature, which prevents causal inference. The lack of metformin and its dosing data represents another constraint, as metformin and its dose-dependent effects on B12 status have been well documented [17-20]. Additionally, potential unmeasured confounders such as dietary habits, genetic polymorphisms, or concomitant medication use may influence the observed relationships. Future research should address these limitations through longitudinal designs with comprehensive metabolic characterization while also exploring the clinical outcomes of B12 repletion in DPN patients.

## Conclusions

Our study demonstrates a high burden of vitamin B12 deficiency (48.2%) among patients with DPN, with a significantly higher prevalence observed in older individuals (54.7% in patients aged 46-60 years) and those with a longer duration of diabetes (72.8% in patients with >5 years of disease). Multivariable logistic regression identified age >45 years and diabetes duration >5 years as independent predictors of B12 deficiency. These findings emphasize the importance of considering vitamin B12 deficiency as a potentially reversible contributor to neuropathy in diabetic patients. Incorporating routine B12 screening, particularly for older individuals and those with prolonged diabetes, into clinical practice may facilitate timely intervention, reduce the risk of progressive neurological deterioration, and improve overall patient care.

## References

[1] Castelli G, Desai KM, Cantone RE (2020). Peripheral neuropathy: evaluation and differential diagnosis. Am Fam Physician.

[2] Zafeiri M, Tsioutis C, Kleinaki Z, Manolopoulos P, Ioannidis I, Dimitriadis G (2021). Clinical characteristics of patients with co-existent diabetic peripheral neuropathy and depression: a systematic review. Exp Clin Endocrinol Diabetes.

[3] Vinik AI, Casellini C, Parson HK, Colberg SR, Nevoret ML (2018). Cardiac autonomic neuropathy in diabetes: a predictor of cardiometabolic events. Front Neurosci.

[4] Sloan G, Shillo P, Selvarajah D (2018). A new look at painful diabetic neuropathy. Diabetes Res Clin Pract.

[5] Djibril AM, Mossi EK, Djagadou AK, Balaka A, Tchamdja T, Moukaila R (2018). Epidemiological, diagnostic, therapeutic and evolutionary features of diabetic foot: a study conducted at the Medico-surgical Clinic, University Hospital Sylvanus Olympio in Lomé (Article in French). Pan Afr Med J.

[6] Ghotaslou R, Memar MY, Alizadeh N (2018). Classification, microbiology and treatment of diabetic foot infections. J Wound Care.

[7] Layden AJ, Täse K, Finkelstein JL (2018). Neglected tropical diseases and vitamin B12: a review of the current evidence. Trans R Soc Trop Med Hyg.

[8] Fritz J, Walia C, Elkadri A (2019). A systematic review of micronutrient deficiencies in pediatric inflammatory bowel disease. Inflamm Bowel Dis.

[9] Miller JW (2018). Proton pump inhibitors, H2-receptor antagonists, metformin, and vitamin B-12 deficiency: clinical implications. Adv Nutr.

[10] Alvarez M, Sierra OR, Saavedra G, Moreno S (2019). Vitamin B12 deficiency and diabetic neuropathy in patients taking metformin: a cross-sectional study. Endocr Connect.

[11] American Diabetes Association (2019). Overview and Update: Standards of Medical Care in Diabetes 2019.

[12] Dagang DJ, Diestro JD, Hamoy-Jimenez G, Isip-Tan IT, Reyes JPB (2016). Validation of the Filipino-translated version of the Michigan neuropathy screening instrument among Filipino patients with diabetes mellitus seen at the Philippine General Hospital. J ASEAN Fed Endocr Soc.

[13] Owhin SO, Adaja TM, Fasipe OJ, Akhideno PE, Kalejaiye OO, Kehinde MO (2019). Prevalence of vitamin B(12) deficiency among metformin-treated type 2 diabetic patients in a tertiary institution, South-South Nigeria. SAGE Open Med.

[14] Raizada N, Jyotsna VP, Sreenivas V, Tandon N (2017). Serum vitamin B12 levels in Type 2 diabetes patients on metformin compared to those never on metformin: a cross-sectional study. Indian J Endocrinol Metab.

[15] Beulens JW, Hart HE, Kuijs R, Kooijman-Buiting AM, Rutten GE (2015). Influence of duration and dose of metformin on cobalamin deficiency in type 2 diabetes patients using metformin. Acta Diabetol.

[16] Ahmed MA, Muntingh G, Rheeder P (2016). Vitamin B12 deficiency in metformin-treated type-2 diabetes patients, prevalence and association with peripheral neuropathy. BMC Pharmacol Toxicol.

[17] Ko SH, Ko SH, Ahn YB (2014). Association of vitamin B12 deficiency and metformin use in patients with type 2 diabetes. J Korean Med Sci.

[18] de Jager J, Kooy A, Lehert P (2010). Long term treatment with metformin in patients with type 2 diabetes and risk of vitamin B-12 deficiency: randomised placebo controlled trial. BMJ.

[19] Nervo M, Lubini A, Raimundo FV, Faulhaber GA, Leite C, Fischer LM, Furlanetto TW (2011). Vitamin B12 in metformin-treated diabetic patients: a cross-sectional study in Brazil. Rev Assoc Med Bras.

[20] Ting RZ, Szeto CC, Chan MH, Ma KK, Chow KM (2006). Risk factors of vitamin B(12) deficiency in patients receiving metformin. Arch Intern Med.

[21] Alharbi TJ, Tourkmani AM, Abdelhay O (2018). The association of metformin use with vitamin B12 deficiency and peripheral neuropathy in Saudi individuals with type 2 diabetes mellitus. PLoS One.

[22] Iqbal Z, Azmi S, Yadav R (2018). Diabetic peripheral neuropathy: epidemiology, diagnosis, and pharmacotherapy. Clin Ther.

[23] Khawaja N, Abu-Shennar J, Saleh M, Dahbour SS, Khader YS, Ajlouni KM (2018). The prevalence and risk factors of peripheral neuropathy among patients with type 2 diabetes mellitus; the case of Jordan. Diabetol Metab Syndr.

